# The p53 Pathway Encounters the MicroRNA World

**DOI:** 10.2174/138920209788185270

**Published:** 2009-05

**Authors:** Apana Takwi, Yong Li

**Affiliations:** Department of Biochemistry and Molecular Biology and Center for Genetics and Molecular Medicine, School of Medicine, University of Louisville 319 Abraham Flexner Way, Louisville, KY 40202, USA

**Keywords:** MicroRNA, p53.

## Abstract

The p53 protein is a transcription factor that regulates multiple cellular processes in human and other high eukaryotes including cell proliferation, differentiation, cell cycle, and metabolism. The central roles played by p53 in tumor development have drawn extensive studies on p53 activation and inactivation. The regulation of p53 and its pathway, as well as its transactivational targets is of prime importance in the understanding of tumorigenesis. Recently, microRNAs (miRNAs) have been reported to be directly transactivated by p53. Equally, p53 and components of its pathway have been shown to be targeted by miRNA thereby affecting p53 activities. In this review, we focus our discussion on the biological and pathological roles of miRNAs in the p53 pathway.

## INTRODUCTION

p53, also known as protein 53 or tumor protein p53, is a transcription factor that regulates multiple cellular processes in human and other high eukaryotes. In human, the TP53 gene encoding the p53 protein is mutated in ~50% of all human cancers [1, 2]. p53 is activated in response to a myriad of stressors such as DNA-damaging agents, radiation, oxidative stress, and proto-oncogenes [3]. These events usually culminate in activation of ATM kinase which in turn leads to phosphorylation of p53. Activated p53 induces cell cycle arrest, or promotes apoptosis depending on the cellular context and extent of DNA damage. The cellular effects of p53 are through its ability to transativate genes that affect cellular processes such as p21 (G1 arrest), 14-3-3σ (G2 arrest) and PUMA (apoptosis) [4-6]. Trans-repressor activities of p53 have also been reported, with p53 down-regulating genes such as the p53-mediated loss of cyclin-dependent kinase (CDK4) and cyclin E2 which both contribute to cell cycle arrest [7]. Recently p53’s ability to regulate expression of microRNAs (miRNAs) and its expression being regulated by miRNAs brings to light a novel class of genes that are regulated by p53. Components of the p53 pathway have also been reported to be targeted by miRNAs.

The first known miRNA, lin-4, was discovered in 1993 by Victor Ambros and colleagues through their study of the heterochromic gene lin-14 in worms. However, it was not until the turn of the millennium that work on miRNAs began in earnest, aiming at elucidating their roles in regulating protein expression. miRNAs constitute an abundant family of endogenously expressed 20-25 nucleotides RNAs that can base-pair to target messenger RNA (mRNA), inhibiting their translation. miRNA-encoded genes are transcribed by RNA polymerase II or III to yield primary transcripts (pri-miRNA). Pri-miRNAs are processed in the nucleus by the nuclear RNase III enzyme, Drosha, to stem-loop-structured miRNA precursor molecules (pre-miRNA). Pre-miRNA are transported to the cytoplasm where they are processed further by another RNase III enzyme, Dicer, which cleaves off the double-stranded (ds) portion of the hairpin and generates a short-lived dsRNA of about 20-25 nucleotides in length. The resulting duplex is eventually unwound and one of the strands gives rise to the mature miRNA which is incorporated into the miRNA-protein complex RISC. The miRNA direct the RISC complex to a target mRNA 3’-UTR leading to either inhibition of translation or degradation of the target mRNA [8, 9]. It is widely believed that perfect complementarity between the miRNA and mRNA leads to mRNA degradation, while partial complementarity favors translational repression. Moreover, miRNA nucleotide 2-9 at the 5’-end, christen ‘seed’ sequence, is important for targeting [9]. Complementarity between the miRNA ‘seed’ and the target mRNA is required for down-regulation of the mRNA.

The facts that miRNAs can target various transcripts, miRNAs are implicated in diverse processes including cellular differentiation, apoptosis, metabolism and cell proliferation [10]. Research over the last decade has led to the realization that certain miRNAs can be classified as oncogenes or tumor suppressors [11, 12]. In this review we focus on miRNAs that have been implicated to function in the p53 pathway.

### miR-34 Family and Apoptosis

**Fig. (1):**
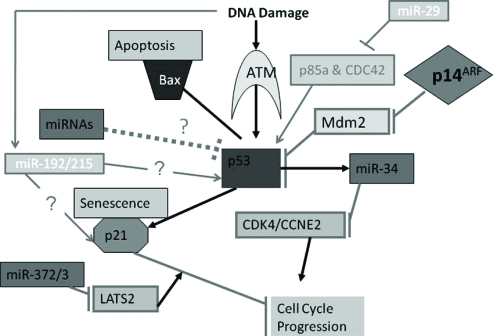
Schematic presentation of miRNAs targeting components of the p53 tumor suppressor network.

In mammals, the miR-34 family members are made up of three miRNAs encoded by two different genes: miR-34b/c (who share a common primary transcript) and miR-34a (encoded on its own. Recent data have shown that genes coding for the miR-34 family are direct transactivational targets of p53 and their over-expression results in the induction of apoptosis, cell cycle arrest and senescence [13-16] (Fig. **1**). Bommer *et al. *[17] showed that miR-34 family members may be tissue specific with miR-34a being expressed at higher level than miR-34b/c with the exception of the lungs. Genome-wide analysis of miRNA expression revealed that miR-34a is most induced following p53 activation [13, 14, 17].

A direct molecular explanation of how miR-34 interferes in the p53 pathway and apoptosis is highlighted in the result published by Lowenstein and colleagues [18]. Their results showed that miR-34a inhibits the Silent Information Regulator 1 (SIRT1) expression (Table **1**), which in turn resulted in increased acethylation of p53 and expression of p21 and Puma. SIRT1 is a NAD-dependent de-acetylase that regulates apoptosis in response to oxidative and genotoxic stress [19]. Recent data suggest that SIRT1 may function as an oncogene and plays a role in tumorigenesis [20] by deactivating p53 through de-acetylation [21]. Lowenstein and colleagues went on to show that SIRT1 led to apoptosis in wild-type p53 human colon cancer cells but not in human colon cancer lacking p53. This indicates a positive feedback loop exist between p53 and miR-34a through an SIRT1-p53 pathway.

Xu *et al. *[30] used a different approach to show that miR-34a plays a central role in the p53 pathway. They showed that in p53-mutant gastric cancer cells, restoration of miR-34a resulted in inhibition of tumorisphere formation and growth. They further demonstrated that the mechanism of miR-34a mediated suppression of self-renewal of gastric cancer cells was related to the modulation of down-stream target of Bcl-2, Notch and HMGA2. This suggests that miR-34a could partially replace p53 in inducing apoptosis or senescence. Taken together, these data indicate that p53 directly activates the expression of miR-34 genes, which play an important role in p53-mediated apoptotic pathway.

### miR-29 Family Regulate p53 Activity

p85α protein is the regulatory subunit of phosphatidylinositol-3 kinase (PI3K) and plays a major role in maintaining the balance of cellular survival and apoptosis. p85α binds to PI3K catalytic subunit p110 and regulates its physiological activity [23]. Park *et al. *[24] showed that p85α has two miR-29 binding sites in it 3’-UTR region. Also, they showed that miR-29 targets CDC42 3’-UTR. CDC42 protein is a member of the Rho family of GTPasas. It regulates cell morphology, cell migration and cell cycle progression [25]. Data gathered by Park *et al. *[24] showed that miR-29 down-regulates CDC42 and p85α, which in turn lead to activation of p53. Over-expression of miR-29 in Hela cells resulted in up-regulation of p53 as well as increased p53-mediated apoptosis. However, over-expression of miR-29 alone, unlike miR-34a, could not lead to apoptosis, but as miR-29 functioning in apoptosis required wild type p53 as apoptosis was not observed when miR-29 was over-expressed in p53-null nor p53 mutant cells. This finding further provides added evidence on the role of miRNA in the p53 pathway and tumorigenesis. Paradoxically, miR-29 acted in a fashion different from miR-34 since it is an apoptotic inducer only in the presence of wild type p53 gene.

### miR-192 and miR-215 Induces p53-Mediated Cell Cycle Arrest

One of the physiological roles of p53 is cell cycle arrest, mediated as a result of p53 transcriptional activation of either p21 (G1 arrest) and/or 14-3-3σ (G2 arrest). In a recent paper published by Dobbelstein and colleagues [26] p53 was shown to induce, together with miR-34a, three additional miRNAs: miR-192, miR-194, and miR-215. These miRNAs increase p53 and p21 protein levels, suppress colony formation, and promote cell cycle arrest. Interestingly, the effect of these miRNAs on colony formation was only partially p53-dependent. Thus, miR-192, miR-194 and miR-215 are regarded as an amplifier to p53 and p53-dependent mediators of cell cycle arrest. However, it is worth noting that increased expression of these three miRNAs was not solely p53-dependent, since p53 is a ubiquitously expressed protein whereas these miRNAs are specifically found in the liver and colon [27]. Therefore, factors other than p53 are responsible for the increase in miR-192 and miR-215 expression level. Chau and colleagues [28] showed that genotoxic stress could lead to up-regulation of miR-192 and miR-215, which in turn lead to a gene expression signature that is highly enriched for regulators of cell cycle. These results imply that miR-192 and miR-215 work in synergy with the p53 pathway in regulating cell cycle arrest and thus they are critical to tumor formation.

### miR-372 and miR-373 can Substitute for Loss of Wild Type (WT) p53

Escaping from oncogene-induced senescence is a pre-requisite for cell transformation. Under oncogenic stress, cells usually arrest excessive cell proliferation by activating the p53 pathway. Agami and colleagues [29] showed that over-expression of the miR-371 cluster was sufficient to overcome cell proliferative arrest following RAS^V12^ introduction. Two miRNAs in this cluster, miR-372 and miR-373 induced cell transformation with oncogenic RAS and WT p53. Their results suggest that miR-272 and miR-373 may participate in the tumorigenesis of tumors with WT p53 and are sensitive to DNA damaging treatment. The Large Tumor suppressor Homolog 2 (LATS2) is a serine kinase whose deletion in flies and mice accelerates cellular proliferation and tumorigenic development [30, 31]. Its over-expression was shown to inhibit cyclin E/CDK2 activity and RAS mediated transformation [32]. It was reported that LATS2 down-regulation stimulates reduplication (same as cyclin E over-expression) [33]. Agami and colleagues were able to show that miR-372 and miR-373 neutralize p53-mediated CDK inhibition, possibly through direct inhibition of the expression of the tumor-suppressor LATS2.

### Other miRNAs that Regulate p53 Activity

The miR-17-92 cluster has been shown to result in down-regulation of Bim [34]. Sawada laboratory [35] showed that overexpression of miR-17-92 resulted in upregulation of c-Myc, decreased expression of p21 that was independent of p53 expression. This result showed that certain miRNA could affect important components of the p53 pathway such as Bim and subsequently result in apoptosis independently of p53.

Kosik *et al. *[36] demonstrated miR-21 directly targets two components in the p53 network. The first one is HNRPK, which can stabilize p53 protein level by interfering with MDM2 and/or act as p53 transcriptional co-activation; and the second is TAp63, which is an isoform of p63, a homolog of p53, and possesses a transactivation domain at the N terminus and are able to transactivate a set of genes, including some targets downstream of p53. These results suggest that miR-21 is an oncogene as it functions against p53 or its target gene activity.

## CONCLUSION

Recent progresses on miRNAs in the p53 pathway have demonstrated that the p53 tumor suppressor network cross-talks with the miRNA regulation system. Prominent among them is the finding that miR-34 family is a direct transactivational target of p53 and it induces apoptosis, cell cycle arrest and senescence [12-15]. The miR-34 story set the precedence and a number of papers have now been published showing that other miRNAs interfere the p53 pathway, in a p53-independent manner (miR-34), partial p53-dependent manner (miR-192, miR-194, miR-215, and miR-21) or a p53-dependent manner (miR-29). Furthermore, miRNAs is implicated in every aspect of cellular outcome of p53 activation: apoptosis (miR-34 and miR-29), cell cycle arrest (miR-192, miR-194, and miR-215), and senescence (miR-34). These exciting results prompt us to contemplate the use of miRNAs in therapeutics against cancer, yet much work is needed to further elucidate the intricacy of the interaction between miRNAs and the p53 pathway.

## Figures and Tables

**Table 1.** miRNAs Function in the p53 Pathway and their Target Genes

miRNAs	Targets	Ref.

miR-34	Cyclin E2 (CCNE2)	[14]
	Cyclin dependent	
	kinase 4 (CDK4)	
	Hepatocyte growth factor receptor (MET)	

	Silent Information Regulator 1 (SIRT1)	[18]

	Bcl-2, Notch and HMGA2 downstream target	[22]

miR-29	p85a	[24]
	CDC42	

miR-192/194/215	Direct target – unknown	[26]
	Indirect target – up-regulate p53 and p53	

miR-372/373	Large Tumor suppressor Homolog 2 (LATS2)	[29]

miR-17-92	Direct target – Bim	[35]
	Indirect target – p21 (up), c-Myc (down)	

miR-21	p63	[26]
	HNRPK	
